# Cardiac Progenitor Cells and Adipocyte Stem Cells from Same Patients Exhibit In Vitro Functional Differences

**DOI:** 10.3390/ijms23105588

**Published:** 2022-05-17

**Authors:** Anthony Soonseng Yee-Goh, Atsushi Yamauchi, Isabelle van Hout, Jayanthi Bellae Papannarao, Ramanen Sugunesegran, Dominic Parry, Philip Davis, Rajesh Katare

**Affiliations:** 1 Department of Physiology, HeartOtago, Dunedin School of Medicine, University of Otago, Dunedin 9010, New Zealand; gohan158@student.otago.ac.nz (A.S.Y.-G.); nishigo42yamauchi6@fuga.ocn.ne.jp (A.Y.); isabelle.vanhout@outlook.co.nz (I.v.H.); jaya.bellaepapannarao@otago.ac.nz (J.B.P.); 2 Department of Cardiothoracic Surgery, Dunedin School of Medicine, University of Otago, Dunedin 9010, New Zealand; ramanen.sugunesegran@postgrad.otago.ac.nz (R.S.); dominic.parry@southerndhb.govt.nz (D.P.); philip.davis@southerndhb.govt.nz (P.D.)

**Keywords:** cardiac progenitor cells, adipocytes stem cells, in vitro cardiac repair, apoptosis, tube formation, ischemic heart disease, stem cells therapy

## Abstract

Cardiac progenitor cells (CPCs) and adipocyte stem cells (ASCs) are widely tested for their efficacy in repairing the diseased heart with varying results. However, no study has directly compared the functional efficacy of CPCs and ASCs collected from the same patient. CPCs and ASCs were isolated from the right atrial appendage and epicardial adipose tissue of the same patients, using explant culture. The flow cytometry analysis confirmed that both the cell types express common mesenchymal stem cells markers CD90 and CD105. ASCs, in addition, expressed CD29 and CD73. The wound-healing assay demonstrated that CPCs migrate faster to cover the wound area. Both cell types were resistant to hypoxia-induced cell death when exposed to hypoxia and serum deprivation; however, the ASCs showed increased proliferation. Conditioned medium (CM) collected after culturing serum-deprived CPCs and ASCs showed differential secretion patterns, with ASC CM showing an increased IGF-1 level, while CPC CM showed an increased FGF level. Only CPC CM reduced hypoxia-induced apoptosis in AC-16 human ventricular cardiomyocytes, while vascular network formation by endothelial cells was comparable between CPC and ASC CM. In conclusion, ASCs and CPCs exhibit differential characteristics within the same patient, and in vitro studies showed that CPCs have marginally superior functional efficacy.

## 1. Introduction

The potential for cell therapy to regenerate the ischemic heart has been established in a multitude of preclinical and clinical studies. Many stem cell types have been studied with varying levels of effectiveness in repairing the diseased heart [1]. However, very few studies have directly compared the functional properties of stem cell populations collected from the same patient, and the ideal stem cell candidate for cardiac repair is yet to be identified.

Cardiac progenitor cells (CPCs) [2] and adipose-derived stromal cells (ASCs) [3] show the potential to promote cardiac repair. Cardiac progenitor cells (CPCs) have been shown to have therapeutic potential, as they can differentiate directly into cells of cardiovascular lineage, such as cardiomyocytes, endothelial cells and smooth muscle cells. They also secrete paracrine factors that promote cell survival and neovascularization and reduce inflammation and fibroblast differentiation [4]. ASCs, on the other hand, also secrete paracrine factors with a similar therapeutic effect on the heart. In particular, ASCs have been demonstrated to have the potential to induce angiogenesis [5,6]. ASCs also have the advantage of being isolated in great numbers through a relatively straightforward procedure. Thus, both ASCs and CPCs present as potential candidates for cardiac repair. In addition, these cells are similar in size, and both show a spindle-shaped morphology [7,8]. Both cell types have also shown benefits in clinical trials, further showing their potential for use in IHD [9,10,11]. However, no studies compared the functional properties of ASCs and CPCs collected from the same patient. Comparing cells collected from the same patient is particularly important to account for patient factors that can affect cell characteristics.

In this study, we compared the paracrine effects of CPCs collected from the right atrial appendage (RAA) tissue and ASCs collected from epicardial adipose tissue (EAT) of the same patient on in vitro angiogenesis migration and apoptosis. While both CPCs and ASCs demonstrated therapeutic effects, CPCs had superior effects on migration and survival under hypoxic conditions. Hence, our study has provided the first evidence that there could be a functional difference between CPCs and ASCs collected from the same heart; thus, it is crucial to determine the cell type based on the type of the disease.

## 2. Results

### 2.1. Characterization of Cells Derived from RAA and EAT

The cells started to migrate from the explant within 3 to 4 days and reached confluency in 10 days. The images showed a spindle-shaped morphology (Supplementary Figure S1), an identical feature of the mesenchymal stem cells (MSCs).

**Figure 1:**
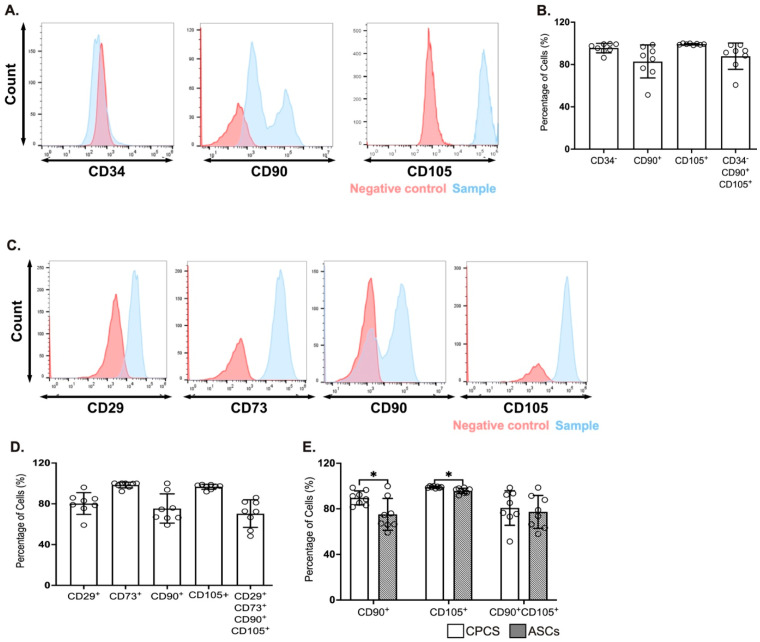
Surface markers for CPCs and ASCs. (**A**,**C**) Representative histograms showing the expression of CPC markers (CD34, CD90 and CD105) (**A**) and ASC markers (CD29, CD73, CD90 and CD105) (**C**); (**B**,**D**) Quantitative scatter plot column graph showing the percentage of CPC (**B**) and ASC (**D**) surface markers. (**E**) Quantitative scatter plot column graph showing the percentage of cells expressing the established MSC markers CD90 and CD105; * *p* < 0.05. Data are represented as mean ± SEM (n = 8 in each group).

The CPCs were identified by the expression of CD34, CD90 and CD105 [4,12]. A majority of the cells were negative for CD34 (95.5 ± 1.6%; see Figure 1A,B), confirming they were resident in the heart. They also showed strong positivity for the established CPC marker CD105 (99.1 ± 0.7%; see Figure 1A,B), while they were heterogeneous for CD90 (82.3 ± 1.6%; see Figure 1A,B). This is also reflected in the proportion of cells that were negative for CD34 and positive for both CD90 and CD105 (87.8 ± 4.4%; see Figure 1B).

The ASCs were characterized for the expression of CD29, CD73, CD90 and CD105 [13,14]. A majority of cells were positive for CD29 (80.2 ± 3.7%; see Figure 1C,D), CD73 (98.3 ± 0.9%; see Figure 1C,D) and CD105 (96.6 ± 0.9%; see Figure 1C,D), while heterogeneous for CD90 (75.4 ± 5.1%; see Figure 1C,D). Similar to CPC, the percentage of cells that were positive for all the four markers was dependent on CD90 expression (70.3 ± 4.8%; see Figure 1D).

A comparison of surface markers between CPCs and ASCs showed a significantly higher expression of CD90 (*p* = 0.04) and CD105 (*p* = 0.01) in CPCs compared to ASCs (Figure 1E). However, CD90 and CD105 double-positive cells were comparable between both cell types (Figure 1E).

### 2.2. CPCs Migrate Faster than ASCs

**Figure 2:**
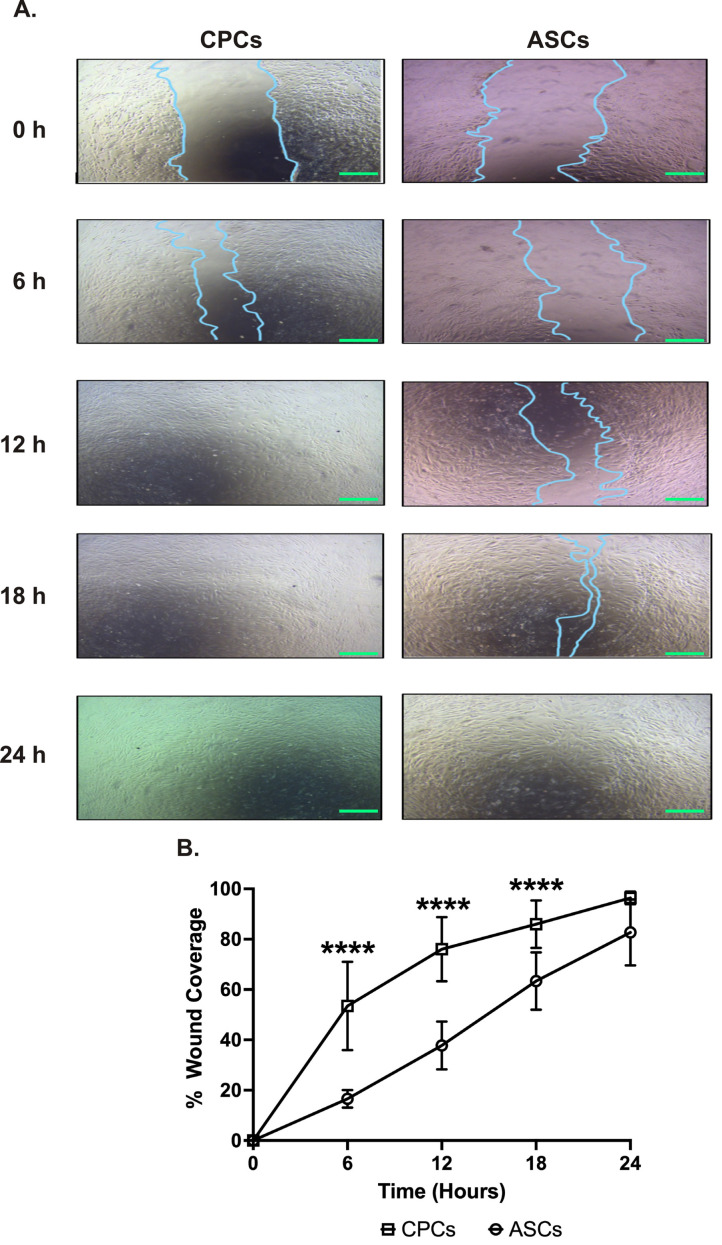
CPC migrates faster. (**A**) Representative images showing the migration of CPCs and ASCs to cover the wound. Images are taken at different time points at 40× magnification. Scale bars, 500 µm. (**B**) Line graph showing the percentage wound coverage over the 24 h experimental period. Data are represented as percentage of wound coverage and are mean ± SEM; n = 8 in each group; **** *p* < 0.0001.

The wound-healing assay was performed to compare the migration between cell types over 24 h. The serial analysis showed that CPCs could migrate faster starting from 6 h. CPCs covered almost 80% of the wound area in 12 h, while ASCs covered only 40% of wound coverage (78 ± 16% in CPCs vs. 38 ± 9% in ASCs, *p* < 0.0001; see Figure 2A,B). Interestingly, at 24 h, both cell types were able to almost completely cover the wound with no significant difference in coverage between ASCs and CPCs at this time point (96 ± 3% in CPCs vs. 83 ± 13% in ASCs, *p* = 0.07; see Figure 2A,B).

### 2.3. CPCs and ASCs Were Resistant to Hypoxia-Induced Apoptotic Cell Death

**Figure 3:**
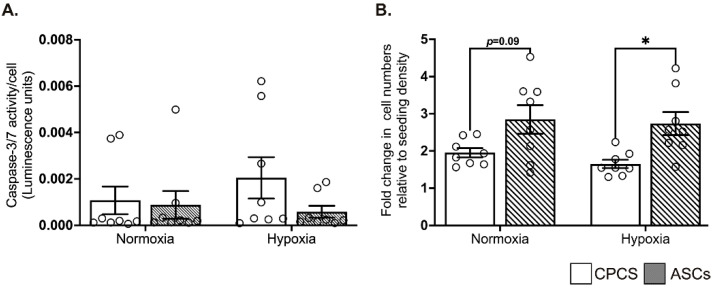
Effect of hypoxia on CPCs’ and ASCs’ quantitative scatter plot column graphs showing caspase-3/7 activity (**A**) and cell proliferation (**B**) in CPCs and ASCs cultured under normoxia and hypoxia. Caspase-3/7 activity is expressed after normalizing to the cell number (**A**). Cell proliferation is expressed as fold changes in cell numbers relative to the seeding density (**B**). Data are represented as mean ± SEM (n = 8 in each group); * *p* < 0.05. All the experiments were performed in triplicates and repeated at least 2 independent times.

When exposed to hypoxia, compared to the cardiomyocytes, both the CPCs and ASCs showed comparable resistance to the activation of pro-apoptotic caspase-3/7, suggesting the ability of both cell types to survive under ischemic conditions (Figure 3A).

### 2.4. ASCs Showed Better Proliferation in Serum-Deprived Normoxic and Hypoxic Conditions

Under serum-deprived normoxic and hypoxic conditions, ASCs tended to show a higher proliferation than CPCs in both normoxia (1.9 ± 0.3 in CPC vs. 2.8 ± 1 in ASC, *p* = 0.09; see Figure 3B) and hypoxia (1.7 ± 0.3 in CPC vs. 2.7 ± 0.9 in ASC, *p* = 0.03; see Figure 3B). However, contrary to the other studies, we did not observe any increase in proliferation when the cells were exposed to hypoxia. Furthermore, there was no significant difference in proliferation between normoxia and hypoxia within each cell type (Figure 3B).

### 2.5. Differential Secretion of Paracrine Cytokines by CPCs and ASCs

**Figure 4:**
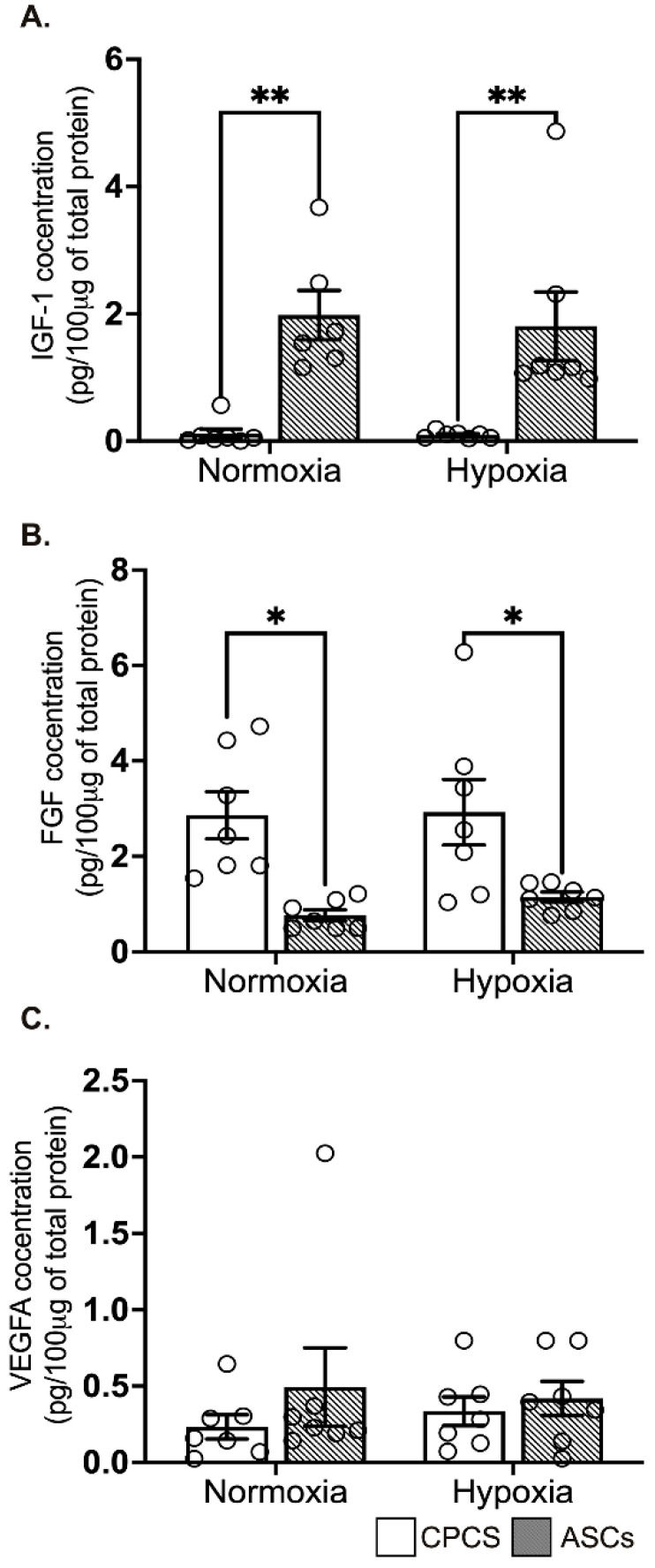
Secretion of paracrine factors by CPCs’ and ASCs’ quantitative scatter plot column graphs showing the level of insulin growth factor-1 (IGF-1) (**A**), fibroblast growth factor (FGF) (**B**) and vascular endothelial cell growth factor (VEGF) (**C**) measured by ELISA in the CM collected from CPCs and ASCs cultured under normoxia and hypoxia. Data are represented as mean ± SEM (n = 6 in each group); * *p* < 0.05 and ** *p* < 0.01. All the experiments were performed in duplicates.

The concentration of secreted IGF-1 levels was significantly higher in ASCs compared to CPCs both in normoxia (0.1 ± 0.2 pg/µg of protein in CPC vs. 1.9 ± 0.9 pg/μg of protein in ASC, *p* = 0.004; see Figure 4A) and hypoxia (0.1 ± 0.1 pg/μg of protein in CPC vs. 1.8 ± 1.4 pg/μg of protein in ASC, *p* = 0.006; see Figure 4A), while secreted FGF was significantly higher in CPCs both in normoxia (2.9 ± 1.3 pg/μg of protein in CPC vs. 0.8 ± 0.3 pg/μg of protein in ASC, *p* = 0.01; see Figure 4B) and hypoxia (2.9 ± 1.8 pg/μg of protein in CPC vs. 1.1 ± 0.3 pg/μg of protein in ASC, *p* = 0.04; see Figure 4B). However, pro-angiogenic factor VEGF-A concentration was comparable between both the cell types in normoxia (0.2 ± 0.2 pg/100 μg of protein in CPC vs. 0.5 ± 0.7 pg/100 μg of protein in ASC, *p* = 0.6; see Figure 4C) and hypoxia (0.3 ± 0.2 pg/100 μg of protein in CPC vs. 0.4 ± 0.3 pg/100 μg of protein in ASC, *p* = 0.9; see Figure 4C). Interestingly, we did not see any significant difference between normoxia and hypoxia in both the cell types.

### 2.6. CPC CM Significantly Reduced Hypoxia-Induced Apoptotic Cell Death in AC-16 Cardiomyocytes

**Figure 5:**
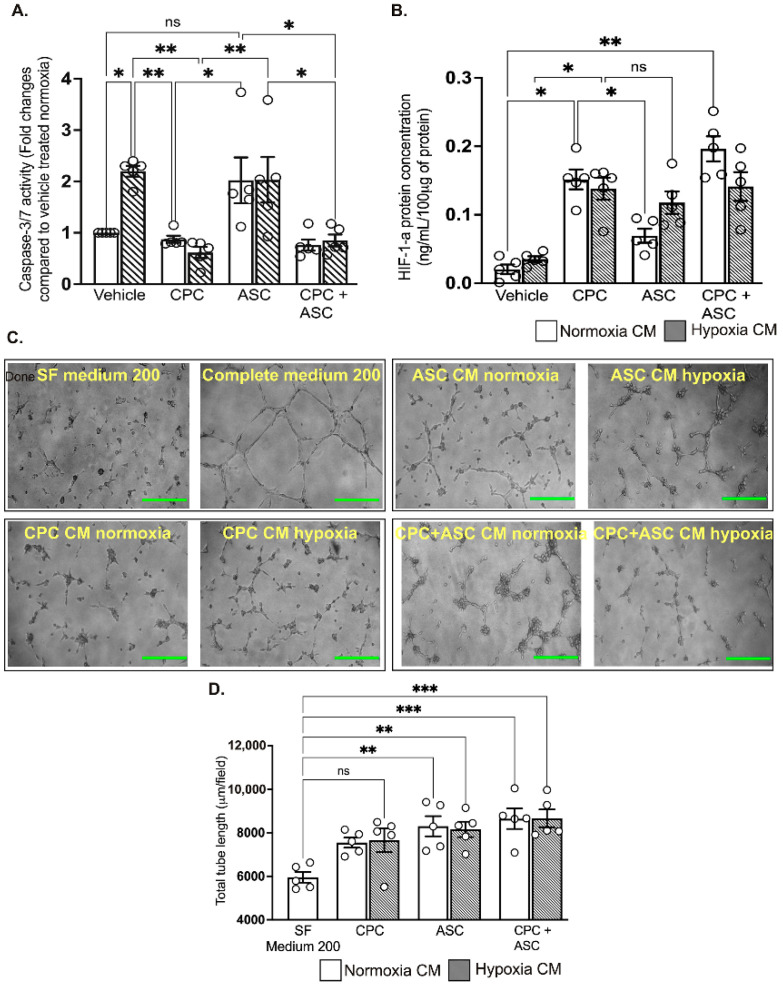
Functional effects of CM on cardiomyocytes and endothelial cells A&B. Quantitative scatter plot column graphs showing caspase-3/7 activity (**A**) and HIF-1α level; ((**B**), measured by ELISA) in AC16 ventricular cardiomyocytes exposed to hypoxia after treatment with either vehicle or CM collected from CPCs and ASCs cultured under normoxia and hypoxia. Caspase-3/7 activity was normalized to the cell number and represented as fold change to vehicle treated group in normoxia (n = 5 patients, quintuplicates). HIF-1α is expressed as the concentration per 100 ug of protein used for the assay (n = 5 patients, duplicates). Data are mean ± SEM. (**C**) Representative phase contrast images showing tube formation in HUVECs. Scale bars, 500 µm. (**D**) Quantitative scatter plot column graph showing the total tube length (n = 5 patients, triplicates, 2 independent repeats). Data represented as mean (SEM) μm/field; * *p* < 0.05, ** *p* < 0.01 and *** *p* < 0.001. ns—non significant.

To determine the pro-survival effects of the paracrine factors secreted in the CM, AC-16 cardiomyocytes were treated with CM collected from CPCs and ASCs cultured under normoxic and hypoxic conditions. Despite increased pro-survival IGF-1 concentration, ASC CM could not reduce pro-apoptotic caspase-3/7 activity in AC-16 cardiomyocytes that are grown under hypoxic conditions (see Figure 5A). In contrast, CPC CM significantly reduced caspase-3/7 activity of cardiomyocytes irrespective of their collection from either normoxia and hypoxia culture conditions (*p* = 0.008, vehicle-treated group vs. normoxic CPC CM; and *p* = 0.004, vehicle-treated group vs. hypoxic CPC CM; see Figure 5A). Combined treatment with CPC and ASC CM did not show any additive effects on caspase-3/7 activity (Figure 5A). As CPC CM did not show any change in the level of IGF-1, we measured whether activation of another pro-survival factor such as HIF-1α in the cardiomyocytes could have reduced caspase-3/7 activity. ELISA confirmed significant activation of HIF-1α in the cardiomyocytes that were treated with both normoxia (0.16 ± 0.03 ng/100 μg of protein in CPC CM vs. 0.02 ± 0.02 ng/100 μg of protein in vehicle, *p* = 0.01; see Figure 5B) and hypoxia (0.14 ± 0.04 ng/100 μg of protein in CPC CM vs. 0.04 ± 0.04 ng/100 μg of protein in vehicle, *p* = 0.01; see Figure 5B) CPC CM. While there was an increase in HIF-1α levels in cardiomyocytes treated with hypoxia ASC CM (0.12 ± 0.04 ng/100 μg of protein in ASC CM; see Figure 5B), this was not significant (*p* = 0.05 vs. vehicle). As with the caspase-3/7 activity, there were no synergistic effects in the level of HIF-1α following the combination treatment (Figure 5B).

### 2.7. Both CPC and ASC CM Enhanced Tube Formation of HUVECs

Finally, to determine the pro-angiogenic effects of the paracrine factors secreted in the CM, HUVECs seeded on Matrigel were treated with CM collected from CPCs and ASCs. Compared to treatment with SF medium 200, all other treatments significantly increased tube formation (*p* < 0.05; see Figure 5C,D and Supplementary Figure S2), with no difference in the CM collected from either CPC or ASCs. There was no synergistic effects on tube formation with the combination treatment (Figure 5C,D).

## 3. Discussion

To our knowledge, this is the first study comparing the characteristics and functional properties of CPCs and ASCs isolated from the same patient. Our results showed that CPCs and ASCs express common MSC markers—although there was a difference in functional characteristics with CPCs can migrate faster, possibly due to increased CD90 and FGF secretion—while ASCs proliferate better. Further, the pro-survival paracrine factors secreted by both cells differed, with ASCs secreting more IGF-1. However, this did not reflect in the functional studies where only the CM collected from CPCs reduced activation of pro-apoptotic caspase-3/7 by the cardiomyocytes exposed to hypoxia, which was likely due to the activation of the master transcriptional factor HIF-1α.

We chose to use a heterogeneous population rather than a purified single cell population used by other studies, as this mimics the best in vivo situation, where most of the cells are heterogeneous. In addition, the low proportion of CD34 positive cells in both CPCs and ASCs suggested that a majority of our cells were resident in the RAA tissue and are devoid of cell types that migrated to the heart [15].

Migration is a critical characteristic of cell therapy, as cells must migrate to the injury site to mediate their therapeutic effects [16]. While we expected similar levels of migration for both cell types, CPCs showed significantly higher wound coverage than ASCs. This could be partially due to the increased expression of CD90 in CPCs. CD90 also has a role in cell migration and adhesion. Hence a higher expression of CD90 in CPCs could have helped in better migration properties [17]. In addition, studies have also demonstrated that the secretion of paracrine factors also plays a role in migration. For example, cytokines such as tumor necrosis factor-alpha (TNF-α), VEGFA, PDGF, FGF and hepatocyte growth factor (HGF) and chemokines such as stromal cell-derived factor-1 (SDF-1) and monocyte chemoattractant proteins (MCP) can increase the migration potential of the cells [16]. While we did not measure all of these factors in our study, CPCs showed higher secretion of FGF. Furthermore, our previous studies showed increased secretion of HGF in CPCs [4,18]. Therefore, a combination of increased CD90, FGF and HGF may have increased the migration potential of CPCs. This suggests that CPCs may have improved potential following transplantation, due to their ability to migrate faster to the ischemic area.

In order to be effective for therapy, transplanted cells must be able to survive and proliferate under ischemic conditions [4,19]. Our results confirmed that both the cell types could survive the ischemic conditions, while ASCs were also able to proliferate more. This could be again related to the secretion of paracrine factors and, in particular, secretion of IGF-1 [20,21]. Previous studies have shown that IGF-1 has mitogenic effects by promoting G1/S cell cycle progression through Akt-dependent and tyrosine kinase–signaling pathways [22,23]. These signaling pathways activate cell cycles and antiapoptotic pathways. Of note, IGF-1 was significantly higher in ASCs irrespective of culture conditions. Contrary to the other studies, we did not observe any significant increase in the proliferation of stem cells under hypoxic condition [24,25]. While the exact reason is unclear, other studies showed that varying lengths of culture time could affect proliferation, with longer culture periods showing more significant differences in proliferation in normoxic and hypoxic conditions [26,27]. As the current study is limited to 24 h, it may be worthwhile to test the effect of long-term culture on proliferation. This is also evident by the lack of any increase in IGF-1 and VEGF-A secretion and activation of pro-survival and -angiogenic genes in response to hypoxia in our study. One other possibility may be due to the age of the donors. ASCs derived from older donors displayed lower proliferation due to higher levels of DNA methylation compared [28,29,30]. As both ASCs and CPCs in this study are derived from patients older than 60 years of age, this could be another reason for the lack of proliferation response under hypoxic conditions.

We, along with others, have shown that stem cells promote tissue healing through paracrine secretion of growth factors that stimulate the repair and growth of the host cells and the recruitment of endogenous stem cells rather than by differentiation [19,31]. CPC CM reduced hypoxia-induced cardiomyocyte apoptosis in our study, while this effect was not observed with ASC CM. In support of our findings, RAA CPCs have been shown to mediate antiapoptotic effects through the PI3K/AKT and PKCε pathways [19]. Furthermore, cardiomyocytes treated with CPC CM showed increased HIF-1α levels. HIF-1α can be stimulated through the PI3K/AKT pathways [32,33], which activates the pro-survival cascade [34]. This could be the underlying mechanism for reduced apoptotic cell death with CPC CM. Even though ASC CM shows higher caspase 3/7 activity than CPC CM, it is important to note that higher ASC proliferation could potentially increase the cell number to match the higher rates of apoptosis.

In conclusion, results from this study suggest that, although collected from the same patient, ASCs and CPCs exhibit differential functional abilities. CPCs may be marginally superior, as they showed better migration and reduced apoptosis than ASCs, which could proliferate better. CPC CM was also able to activate the pro-survival HIF-1α in cardiomyocytes exposed to hypoxia. Both the cell types showed comparable angiogenic potential. However, these results must be taken in the context of the technical difficulties in procuring CPCs and ASCs, with ASCs being easier to isolate and in higher numbers. Therefore, the choice between CPCs and ASCs may depend on the required effect. The main strength of this study is the isolation of CPCs and ASCs from the same patient, as we avoided the interpatient variations when comparing the two cell types. The study also comes with some limitations. First, all the cells were isolated from patients with IHD, although the severity of the disease is matched between the patients. Second, while we took all the precautions during the preparation and growth of the cells to avoid any CPCs’ contamination in ASC, which also reflected in the flowcytometry analysis showing the expression of ASCs specific markers, we cannot completely exclude some contamination of CPCs. Third, previous studies have shown that long-term culture with a high number of population doublings can lead to a change in phenotype of MSCs [35], although we did not find any evidence of change in phenotypes. Overall, this study has allowed direct comparison of two different cell types highly regarded as having potential for cardiac repair. Further in vivo studies using small and larger animal models is essential to confirm these in vitro findings.

## 4. Methods

### 4.1. Ethical Approval

The human myocardium study was approved by the Health and Disability Ethics Committee of New Zealand (LRS/12/01/001/AM16). All patients were informed and consented to the study before surgery and obtaining the tissue samples.

### 4.2. Tissue Collection

Samples of EAT and RAA were collected from patients (n = 8, Table 1) undergoing on-pump coronary artery bypass graft (CABG) surgery at Dunedin Public Hospital, New Zealand. Patients with diabetes were excluded from this study. All parameters were comparable between each patient, except one patient with a reduced ejection fraction (<50%); however, this sample was not a consistent outlier during the study. EAT and RAA samples were collected in Krebs–Ringer–Henseleit (KRH) solution and kept on ice until explant. To ensure patient homogeneity, patient parameters were checked on the REDCap database to ensure similar characteristics between patients.

### 4.3. Cell Culture

#### 4.3.1. Explant Culture of CPCs and ASCs

After dissecting the vessels or tissue other than adipose tissue for EAT or muscle tissue for RAA, the tissues were washed twice in phosphate-buffered saline (PBS) (Oxoid, Basingstoke, UK). The samples were then cut into pieces of approximately 3 mm^3^ in size. The pieces were then briefly dried on a paper towel, placed on a new 6 cm culture dish and incubated at 37 °C for 30 min. Following incubation, EAT was supplemented with Dulbecco’s Modified Eagle Medium (DMEM) (Gibco, Waltham, MA, USA) with 4.5 g/L glucose and L-glutamine supplemented with 10% FBS (Gibco, Waltham, MA, USA) and 1X antibiotic/antimycotic (anti–anti) was added for EAT. RAA tissues were supplemented with Ham’s F12 complete media containing Ham’s 12 nutrient mixture (Thermofisher Scientific, NZ) supplemented with 10 ng/mL human basic fibroblast growth factor (bFGF), 0.005 U/mL human erythropoietin (EPO), 10% foetal bovine serum (FBS) (Gibco, USA) and 1X anti–anti (Gibco, USA). Tissues were incubated in a 5% CO_2_/95% air-humidified incubator at 37 °C. The media was replaced every alternative day. Cells were passaged when they reached 85–95% confluency. All the experiments were conducted with cells in passages three to six.

#### 4.3.2. AC16 Cardiomyocytes

AC16 cardiomyocytes (Merck Millipore, Burlington, MA, USA) are a proliferating cell line derived from fusing primary cells from adult human ventricular heart tissue with the simian virus (SV) 40 transformed, uridine auxotroph human fibroblasts devoid of mitochondrial DNA [36]. AC16 cells were used to determine the paracrine effects of CPCs and ASCs on cardiomyocytes survival. AC16 cardiomyocytes were cultured in low-glucose DMEM complete media containing DMEM with 1 g/L glucose, L-glutamine, sodium pyruvate and 3.7 g/L sodium bicarbonate pH adjusted to 7.4 (Gibco, USA). This was further supplemented with 10% FBS (Thermofisher Scientific, NZ) and 1X anti–anti (Thermofisher Scientific, NZ).

#### 4.3.3. Human Umbilical Vein Endothelial Cells

Human umbilical vein endothelial cells (HUVECs) (ATCC, USA) are primary endothelial cells derived from human umbilical cord veins. HUVECs were used to test the paracrine effects of CPCs and ASCs on endothelial cells’ migration, proliferation and tube formation. HUVECs were cultured in Medium 200 complete media containing basal Medium 200 (Thermofisher Scientific, NZ) supplemented with 8% FBS, 1X anti–anti and low serum growth supplement (LSGS) (Thermofisher Scientific, NZ) containing basic FGF, human recombinant EGF, FBS, heparin and hydrocortisone.

### 4.4. Flow Cytometry Characterization of ASCs and CPCs

ASCs and CPCs were characterized by using flow cytometry for specific cell markers. Following trypsinization and washing, 1 × 10^6^ cells were resuspended in 50 µL of flow cytometry buffer. Cells were then stained with antibodies against the established surface markers for CPCs and ASCs (Table 1) or compensation controls in the dark for 30 min on ice. Following incubation, the cells were washed twice with flow cytometry buffer (440 g for 5 min), resuspended with 250 µL of flow cytometry buffer and stored on ice in the dark (for immediate acquisition) or fixed in 0.5% formaldehyde solution and stored at 4 °C for no longer than 48 h. Before the acquisition, all tubes were vortexed briefly and loaded onto the Gallios flow cytometer (Beckman Coulter, Indianapolis, IN, USA). Fluorescent light (FL) channels corresponding to each fluorophore were included in the acquisition (Supplementary Table S1). Before data acquisition, PMT voltages and gain were adjusted to ensure that all cell populations were detected and that there was a clear separation between positive and negative populations. For each sample, 5000–10,000 events were acquired. Acquisition data were analyzed by using FlowJo (FlowJo, LLC, Ashlan, OR, USA) software. Compensation control data were used to set up a compensation matrix and applied to each sample before gating. Data are expressed as percentage of cells positive for each marker.

### 4.5. Wound-Healing Assay to Determine the Migration Potential of CPCs and ASCs

Cells were seeded at a density of 2X10^4^ cells/well in a 24-well plate in complete Ham’s F12 or DMEM media for CPCs and ASCs, respectively. After 24 h, a scratch was made vertically in the middle of the well, using a yellow tip pipette. The supernatant was aspirated, and cells were washed twice with 100 µL of PBS to remove any remaining traces of media or cell debris. The cells were then supplemented with 500 µL of medium (Hams’s F12 or DMEM) with only 1% FBS and 1X anti–anti. Images were taken immediately and every 6 h for up to 24 h, using a light microscope (Olympus, Tokyo, Japan) fitted with a camera (Lumenera, USA). Wound closure during the experimental period was analyzed by using Fiji software (NIH, Bethesda, MD, USA). Results were expressed as percentage wound closure.

### 4.6. Exposure of CPCs and ASCs to Hypoxic Conditions and Collection of Conditioned Media

Next, to determine the effect of ischemic hypoxic conditions on the activation and release of the pro-survival cascade by CPCs and ASCs, serum- and growth-factor-deprived cells (1X10^5^ cells/T25 flask) were cultured in hypoxic (1% O_2_) conditions. Cells grown in normoxic conditions (20% O_2_) were used as control. After 72 h, the media from each flask were collected and centrifuged at 300 RCF for 5 min to remove any cellular debris. The supernatant, or conditioned media (CM), was then collected and aliquoted into sterile microcentrifuge tubes and stored in a −80 °C freezer until further use. The cells were then washed with PBS and lysed with TRIzol lysis reagent. The cell lysates were stored in a −80 °C freezer until total RNA isolation.

### 4.7. Apoptosis and Cell Proliferation Assay

To assess the ability CPCs and ASCs to survive and proliferate under ischemic conditions, cells were seeded (5 × 10^3^/well) in triplicates in a 96-well plate and cultured under hypoxic conditions, as described above. At the end of 72 h of hypoxia, the culture medium was aspirated and replaced with 25 µL of PBS and 25 µL of caspase-3/7 substrate. The well contents were then mixed on a plate shaker at 250 RPM for 1 min and incubated at room temperature in the dark for 60 min. Luminescence was then measured by using a plate reader. Following caspase measurement, 50 µL of CyQUANT reagent was added to each well to determine the cell proliferation, and after 5 min of incubation, the fluorescence intensity was measured by using a plate reader set at 480 nm excitation and 520 nm emission. Caspase-3/7 activity was normalized to the cell numbers by CyQuant assay and expressed as fold change compared to the normoxia treated CPCs. Cell proliferation was expressed as the fold change relative to seeding density.

### 4.8. Enzyme-Linked Immunosorbent Assay (ELISA) for Secretion of Pro-Survival and Angiogenic Factors in CM

The effect of hypoxia on the secretion of pro-survival insulin growth factor-1 (IGF-1) and pro-angiogenic vascular endothelial growth factor-A (VEGF-A) [20,21] in collected CM was measured by using commercially available ELISA kits (Elisakit.com, Australia), following the manufacturer’s instructions. The concentrations of IGF-1 and VEGF-A were then normalized to the total protein concentration for each sample.

### 4.9. Pro-Survival Effects of CM on Cardiomyocytes

AC16 human ventricular cardiomyocytes were used to determine the pro-survival effect of the CM collected from CPCs and ASCs by measuring the pro-apoptotic caspase-3/7 activity and pro-survival HIF-1 α.

For caspase-3/7 activity, 5 × 10^3^ cells were plated in a white 96-well plate in low-glucose DMEM complete medium in quintuplicates. After 24 h, the culture medium was replaced with new media containing 50 µL of CM and 50 µL of serum-free low glucose DMEM. For the combined CPC and ASC CM treatment, both CMs were mixed at a ratio of 1:1. Negative controls were supplemented with 100 µL of serum-free low glucose DMEM. Cells were then incubated in 1% O_2_/5% CO_2_ (hypoxia) for 72 h to mimic in vivo ischemic conditions. Cells supplemented with complete DMEM medium and grown in normoxia served as the control. At the end of hypoxia duration, caspase-3/7 activity was measured as above and normalized to the cell numbers measured by CyQUANT assay.

For HIF-1α, 5 × 10^5^ cells/well cells were plated in a 6-well culture plate and underwent treatment similar to the experiments for caspase-3/7 activity. At the end of the culture period, total protein was extracted from the cells, and the concentration of HIF-1α was measured by using the commercially available ELISA kit (HIF-1α Human SimpleStep ELISA kit (Abcam, Cambridge, UK)), following the manufacturer’s protocol.

### 4.10. Pro-Angiogenic Effects of CM on Endothelial Cells

Tube formation assay was used to measure the pro-angiogenic effects of CM on endothelial cells. For this, HUVECs were seeded on a Matrigel matrix (Corning, NY, USA) coated 96-well culture plate at the density of 18,000 cells/well. Prior to seeding, HUVECs were suspended in the mixture of serum-free medium and CM at a ratio of 50:50. Negative control wells were seeded in 100 µL of serum-free (SF) medium 200, and positive control wells were seeded in 100 µL of medium 200 complete media. The plate was then incubated in a 5% CO_2_/95% air-humidified incubator at 37 °C for 24 h, with images taken by using a bright field microscope (Olympus, Japan) at 0, 3, 6, 9, 12, 18 and 24 h time points. Data were analyzed by using Fiji software (NIH, USA).

### 4.11. Statistical Analysis

The statistical analyses were conducted by using GraphPad Prism Software (version 9). Differences in CD90 and CD105 expression between ASCs and CPCs were compared by using paired t-tests. Wound-healing assay results were analyzed by using a two-way analysis of variance (ANOVA) with repeated measures, followed by a Tukey post hoc test. The results were expressed as percentage wound closure. A two-way ANOVA was used to compare the caspase-3/7 activity, cell proliferation and ELISA between each group, followed by a Tukey post hoc test. Caspase activity in CM-treated cardiomyocytes, HIF1-α and total tube length were analyzed by using a one-way ANOVA, followed by a Dunnett’s post hoc test. All data were represented as mean (SEM). Differences with a *p*-value ≤ 0.05 were considered statistically significant.

## Figures and Tables

**Table 1:** Patient characteristics.

Sample Number	Age	Sex	BMI	Diabetes	Hypertension	Ejection Fraction (%)
487	72	F	39.0	N	N	55–60
493	64	F	26.4	N	Y	60
500	69	M	35.1	N	N	53.1
765	61	M	34.4	N	N	57.3
769	70	M	25.7	N	Y	55–60
772	67	F	30.5	N	Y	71.6
779	67	M	27.3	N	N	50.6
782	67	M	34.5	N	Y	45–50

BMI—body mass index.

## Data Availability

All the data are included in the manuscript.

## Supplementary Materials

The following supporting information can be downloaded at: https://www.mdpi.com/article/10.3390/ijms23105588/s1.
