# Supplementary material for: Cardiac Progenitor Cells and Adipocyte Stem Cells from Same Patients Exhibit In Vitro Functional Differences
Source: Int J Mol Sci. 2022 May 17;23(10):5588. doi: 10.3390/ijms23105588 (PMC9141982; doi:10.3390/ijms23105588)
Supplement: Supplementary file 1 [file ijms-23-05588-s001.zip › ijms-1698126-supplementary.pdf]

Supplementary Materials

Figure S1: Light microscopy images of explanted cells

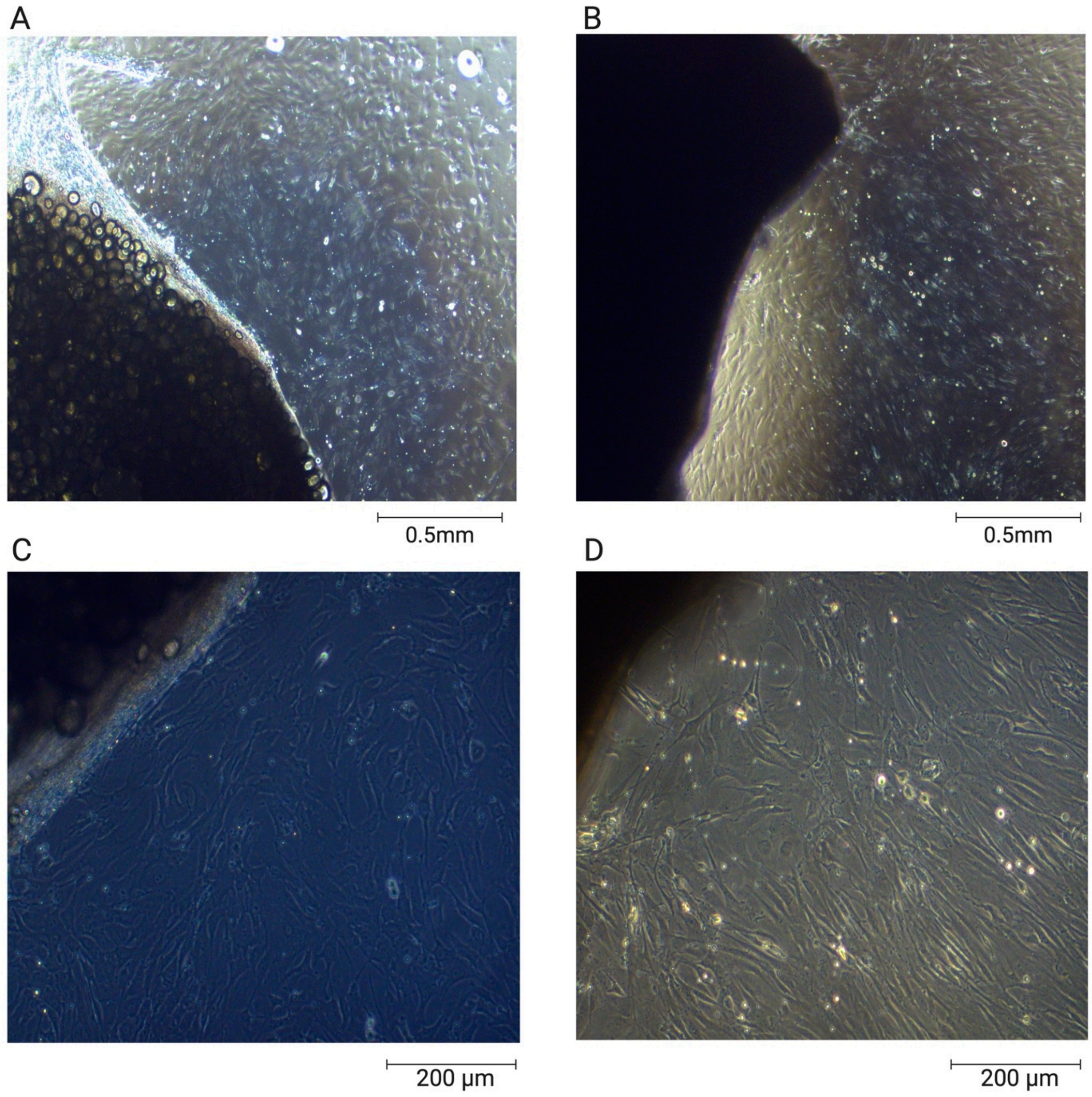

Figure S1: Light microscopy images of explanted cells.

Image showing cells explanted from epicardial adipose tissue (**A&C**) and from right atrial appendage (**B&D**). Inset red squares show a part of the image at higher magnification. Images **A&B** were taken at 40X magnification and **C&D** at 100X magnification.

Supplemental Figure 2

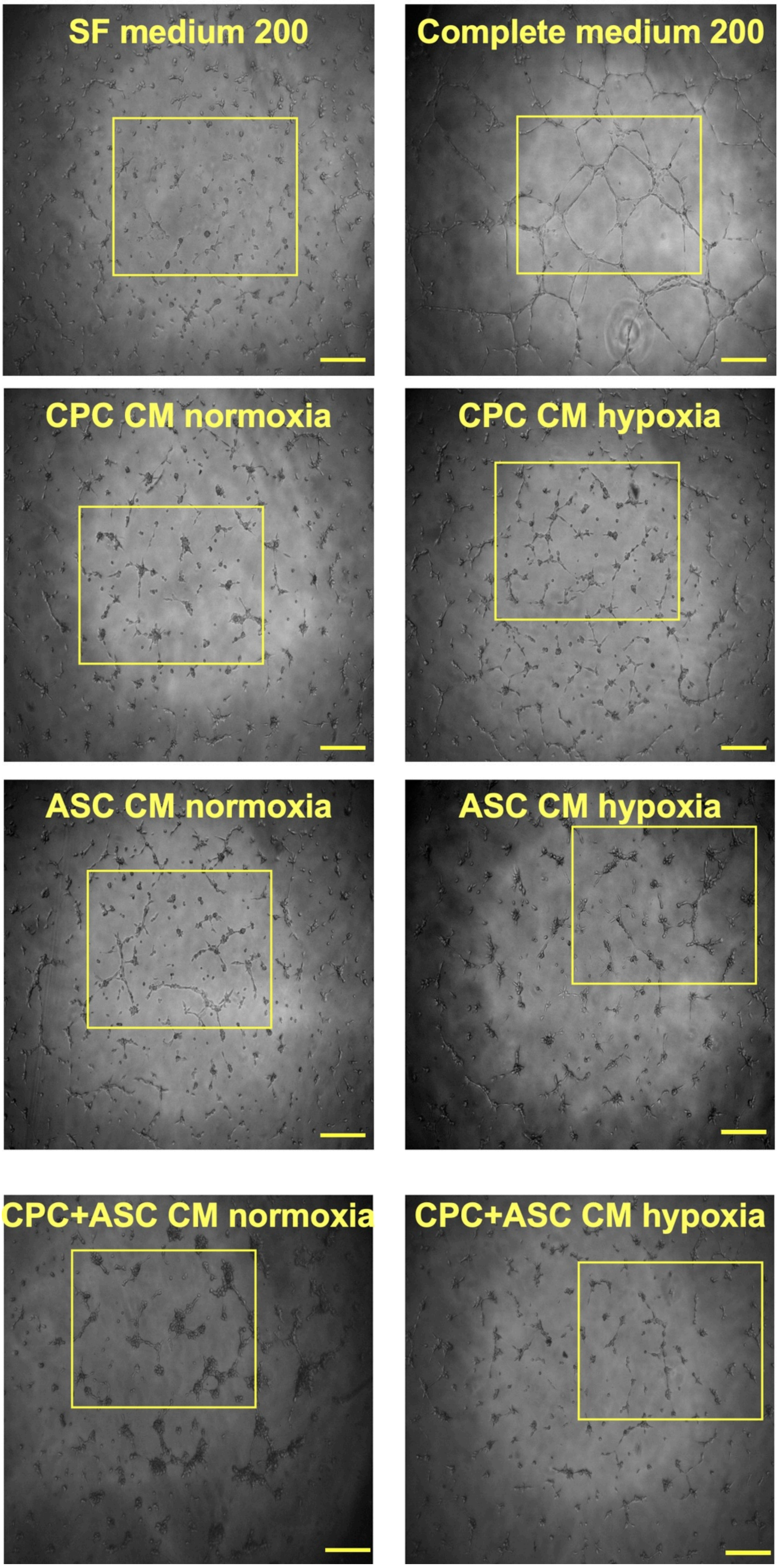

Figure S2: Tube formation assay

Representative full images captured at 40X magnification, showing network formation by HUVECs with or without CM from CPCs and ASCs. Complete medium 200 group is the positive control, while serum free (SF) medium 200 group is the negative control. Boxed area in each image is zoomed in the main manuscript. Scale bars are 500 µm.

Table S1: Table showing relevant fluorescent light (FL) channels and the corresponding fluorophores.

FL Channel	Fluorophore	Conjugated cell marker(s)	Excitation wavelength (nm)	Emission wavelength (nm)
FL1	FITC	CD34/CD29	488	525/40 BP
FL2	PE	CD105	488	575/25 BP
FL5	PE-Cy7	CD73	488	755 LP
FL6	APC	CD90	640	660/20 BP
FL10	ZY	Viability	405	550/40 BP

FL, fluorescent light; BP, band pass; LP, long pass
